# Unveiling novel macrophage-specific biomarkers in MASH through single-cell sequencing for diagnostic modeling

**DOI:** 10.1016/j.jlr.2026.101048

**Published:** 2026-04-28

**Authors:** Tingting Wang, Xiayan Zhu, Yiying Zhang, Lihuang Su, Tongtong Pan

**Affiliations:** 1 Department of Gastroenterology, The First Affiliated Hospital of Wenzhou Medical University, Wenzhou, Zhejiang, China; 2 Wenzhou Key Laboratory of Interdisciplinary and Translational Medicine, The First Affiliated Hospital of Wenzhou Medical University, Wenzhou, Zhejiang, China; 3 Department of Geriatrics, The First Affiliated Hospital of Wenzhou Medical University, Wenzhou, Zhejiang, China

**Keywords:** bioinformatics analysis, carboxypeptidase M, erythropoietin-producing hepatocellular carcinoma receptor B41-like 2, macrophage biomarkers, metabolic dysfunction-associated steatohepatitis

## Abstract

Macrophages are pivotal in the progression of metabolic dysfunction-associated steatohepatitis (MASH), yet their specific markers remain elusive. Herein, we employed an integrated bioinformatics strategy, combining single-cell and bulk transcriptomic data from human MASH livers, to identify macrophage-related differentially expressed genes (Mϕ-DEGs). We pinpointed five core Mϕ-DEGs— *FRMD4B*, *PTK2B*, *CPM*, *SPTLC2*, and *EPB41L2*—that were predominantly expressed in macrophages and enriched within profibrotic M2 subsets. A diagnostic model constructed from these genes demonstrated high accuracy (area under the curve = 0.9865) and was robustly validated in an independent cohort. In human and murine MASH samples, *FRMD4B* and *PTK2B* were consistently downregulated, whereas *CPM*, *SPTLC2*, and *EPB41L2* were upregulated. Protein-level validation by immunohistochemistry and immunofluorescence confirmed these expression patterns in human and mouse livers and in polarized THP-1-derived macrophages. Mendelian randomization analysis identified *CPM* as a significant causal protective factor, suggesting that its upregulation may represent a compensatory response. These genes correlated with altered immune cell infiltration (e.g., T follicular helper and regulatory cells) and were enriched in key MASH pathways, including fatty acid metabolism, sphingolipid signaling, and transforming growth factor beta/PI3K-Akt. Our findings were further corroborated through a multilevel validation framework encompassing clinical samples, a murine MASH model, and in vitro macrophage cultures. This study characterized a robust macrophage-specific gene signature for MASH diagnosis and offered genetic evidence regarding the causal protective role of *CPM*. The pronounced enrichment of these genes in M2 macrophages underscores their critical contribution to immunometabolic dysregulation, offering novel insights into MASH pathogenesis and potential diagnostic and therapeutic targets.

With the increasing prevalence of obesity and metabolic syndrome, metabolic dysfunction-associated steatotic liver disease (MASLD) presents a major global public health challenge, affecting nearly one-third of adults worldwide, particularly in Western countries and China (1, 2). MASLD encompasses a spectrum from simple steatosis to metabolic dysfunction-associated steatohepatitis (MASH), cirrhosis, and hepatocellular carcinoma. MASH represents a critical progressive phenotype, with up to 39% of the patients developing cirrhosis within 10–15 years (1, 2). Despite its clinical significance, effective treatments for MASH remain lacking, underscoring the urgent need to elucidate its mechanisms and identify novel therapeutic targets.

The pathogenesis of MASLD has transitioned from the “second hit” hypothesis to a “multiple-hit” model, involving lipotoxicity, oxidative and endoplasmic reticulum stress, and gut–liver axis dysfunction (3). Within this framework, hepatic macrophages play a crucial role in driving inflammation, fibrosis, and metabolic dysregulation (4). For example, the activation of Kupffer cells and infiltration of monocyte-derived macrophages lead to the sustained release of proinflammatory cytokines and fibrogenic mediators such as transforming growth factor beta 1, which promotes hepatic stellate cell activation and collagen deposition (5). However, the diversity, dynamic polarization, and functional specificity of macrophage subsets in MASH remain poorly characterized.

Advances in high-throughput sequencing and bioinformatics have enabled the systematic dissection of disease-related gene networks. Bulk transcriptomics provides overall gene expression profiles; however, it does not resolve cell-specific signatures and spatial heterogeneity, particularly in low-abundance cell types. Conversely, single-cell RNA sequencing (scRNA-seq) offers unprecedented resolution for exploring cellular diversity and macrophage subsets in the liver (6, 7). Nevertheless, its utility is often limited by technical sparsity and limited sample sizes. Integrating scRNA-seq with multi-omics platforms provides a powerful strategy for uncovering macrophage-related signaling pathways, epigenetic states, metabolic reprogramming, and intercellular crosstalk in MASH (8).

Therefore, in this study, using single-cell and transcriptome sequencing data from patients with MASH and applying bioinformatic approaches, we aimed to identify key macrophage markers, construct related predictive models, and explore relevant molecular mechanisms. Our findings can facilitate the development of new technologies for diagnosing and treating MASH/MASLD.

## Materials and Methods

### Data acquisition

The scRNA-seq dataset GSE212837 (18 liver samples: 3 controls and 15 patients with MASH) was downloaded from the Gene Expression Omnibus database. In this study, we performed a secondary re-analysis of the publicly available gene-cell count matrices and metadata from GSE212837 (9). For bulk transcriptome analysis, series matrix files for GSE164760 (GPL13667) and GSE126848 (GPL18573) were retrieved and used as the training and external validation cohorts, respectively, comprising 110 samples in total (20 controls and 90 disease samples).

### Single-cell RNA-seq data processing and analysis

The same pipeline was applied to the human GSE212837 dataset and the mouse 10× data generated in this study. Using Seurat v5 (10), we filtered cells with nFeature_RNA > 1500, percent.mt < 5, and nCount_RNA < 100,000, which were then normalized with LogNormalize (scale factor = 10,000). The top 2,500 highly variable genes were identified, and principal component analysis was performed. Batch effects were corrected using Harmony (11). Clustering was performed with FindNeighbors and FindClusters and visualized by *t*-SNE (12). Marker genes were identified using FindAllMarkers (|log_2_FC| > 1, adjusted *P* < 0.05, min.pct = 0.25). Cell types were annotated using CellMarker, PanglaoDB, and SingleR (13). For macrophage subpopulations, cells were subset and re-analyzed with 2,000 variable genes, Harmony integration, UMAP, and FindAllMarkers (|log_2_FC| > 0.25, adjusted *P* < 0.05).

### Ligand–receptor interaction analysis (CellChat)

Normalized single-cell expression profiles and cell identity annotations from Seurat were used to construct a CellChat object (14). CellChatDB.human was used as the ligand-receptor reference database, and the analysis focused on the Secreted Signaling category. Overexpressed genes and interactions were identified, communication probabilities were computed, and low-confidence interactions were filtered. Intercellular communication was then quantified by both interaction counts and interaction weights to assess communication frequency and strength among cell types.

### Predictive modeling

From the GSE164760 and GSE126848 datasets, only healthy liver and MASH liver samples were included; MASH-HCC samples were excluded. Macrophage-specific DEGs were identified from scRNA-seq data (|log_2_FC|>1, adj.*P* < 0.05) and matched to bulk datasets. A LASSO logistic regression model (glmnet, alpha = 1, 10-fold CV) was built. The five genes with nonzero coefficients at lambda.min were retained, balancing performance and parsimony. Risk score = Σ(standardized expression × coefficient). Model performance was evaluated using receiver operating characteristics (ROC) and area under the curve (AUC) in training (GSE164760) and validation (GSE126848) sets.

### Mendelian randomization analysis

Summary statistics for MASH were obtained from the FinnGen consortium (R12; outcome ID: finngen_R12_MASH). Using the TwoSampleMR R package, instrumental variables (IVs) for the exposure were selected via extract_instruments at *P* < 1 × 10^−5^, followed by linkage disequilibrium clumping (*R*^2^ < 0.001, window = 10,000 kb) and retention of SNPs with *P* < 5 × 10^−5^. The outcome data for these IVs were then extracted using extract_outcome_data.

The primary analysis employed the inverse-variance weighted (IVW) method, supplemented by sensitivity analyses (MR-Egger, weighted median, and weighted mode). Horizontal pleiotropy and heterogeneity were assessed via the MR-Egger intercept test and Cochran's Q statistic, respectively.

### Correlation analysis of macrophage-related differentially expressed genes with lipid pathways and M2 markers

Gene set variation analysis (GSVA) was performed using the gsva R package to quantify pathway activity scores for each sample in the GSE164760 dataset. Specifically, single-sample gene set enrichment analysis (ssGSEA) was applied with the method parameter set to "ssgsea." The gene sets included fatty acid degradation, linoleic acid metabolism, retinol metabolism, steroid hormone biosynthesis, and steroid hormone metabolism, obtained from the MsigDB database (version 7.0). Pearson correlation coefficients between the expression of each macrophage-related differentially expressed gene (Mϕ-DEG) and the ssGSEA scores of these lipid-related pathways were calculated using the cor.test function in R, with *P* < 0.05 considered statistically significant.

To evaluate the association between Mϕ-DEGs and M2 polarization, we performed Pearson correlation analysis between the expression levels of the five Mϕ-DEGs and established M2-related genes (*ARG1*, *IL10*, *MRC1*, *RETNLA*, as well as M2-associated functional genes such as *CTSB*, *CTSD*, *CSF1R*, and *VEGFA*) in the GSE164760 dataset. Correlation coefficients (R) and *P* values were computed using the cor.test function. Heatmaps and scatter plots were generated using the pheatmap and ggplot2 R packages, respectively.

### Immune infiltration analysis

Immune infiltration was estimated using the ssGSEA implemented during GSVA (method = 'ssgsea', kcdf = 'Gaussian', abs.ranking = TRUE). Differences in immune cell scores between groups were assessed using the Wilcoxon rank-sum test. Correlations between key gene expression and immune cell infiltration were evaluated using Pearson correlation analysis, with *P* < 0.05 considered statistically significant.

### Regulatory network analysis of key genes and transcription factor (TF)–mRNA–miRNA network construction

TFs were predicted using the R package “RcisTarget.” The AUC was calculated for each motif–gene set pair to estimate motif overexpression. The normalized enrichment score for a motif depends on the total motifs in the database and their AUC distribution within the gene set. Key gene-associated miRNAs were sourced from the miRcode database, and their networks were visualized using Cytoscape software.

### Liver tissue and clinical data of patients with MASH

This study was conducted in accordance with the principles of the Declaration of Helsinki and approved by the Ethics Committee of The First Affiliated Hospital of Wenzhou Medical University (protocol code: 2020-zz-028). Patients provided informed consent. Liver tissues were obtained via biopsy from patients with MASH (n = 21), whereas adjacent normal liver tissues were obtained via surgical dissection from patients with hepatoma (n = 18).

### Establishment of a high-cholesterol and high-fat-diet-induced MASH model in mice and histopathologic assessment of damage

Seven-week-old male C57BL/6 mice were sourced from Vital River Laboratory Animal Technology Co., Ltd. Animal experiments followed the guidelines of the Institutional Animal Care and Use Committee of Wenzhou Medical University (No. WYDW2018-0252). Mice were fed a high-cholesterol and high-fat diet (CL, 60% kcal from fat, #D06061403; Research Diets) or normal chow (NC, #CRF-1; Charles River Laboratories) for 12 weeks, as previously reported (15). Blood was centrifuged at 1,500 × *g* for 15 min to obtain serum, which was analyzed for triglycerides, total cholesterol, aspartate aminotransferase, alanine aminotransferase, interleukin (IL)-1β, and tumor necrosis factor (TNF)-α using an autochemistry analyzer (#AU5800; Beckman Coulter).

### Histopathological and immunohistochemical staining

Paraffin-embedded liver sections (4 μm) were deparaffinized, rehydrated, and subjected to H&E or Oil Red O staining, followed by microscopic examination (Nikon). For immunohistochemistry (IHC), antigen retrieval was performed in a citrate buffer (pH 6.0) at 95°C for 20 min, and endogenous peroxidase activity was blocked with 3% H_2_O_2_ for 10 min. Sections were incubated overnight at 4°C with the following primary antibodies: anti-CD68 (#ab213363, 1:8000, Abcam), anti-FRMD4B (#bs-8236R, 1:200, Bioss Inc.), anti-PTK2B/PYK2 (#bsm-52553, 1:500, Bioss Inc.), anti-CPM (#DF3899, 1:200, Affinity Biosciences), anti-SPTLC2 (#DF12231, 1:200, Affinity Biosciences), and anti-EPB41L2 (#15437-1-AP, 1:200, Proteintech). After washing with PBS, sections were incubated with an HRP-conjugated goat anti-rabbit secondary antibody (#ab205718, 1:500, Abcam) for 1 h at room temperature. Signals were developed using a DAB substrate (#P0202, Beyotime Biotech Inc.), counterstained with hematoxylin, and imaged using a light microscope (Nikon). Staining intensity was quantified using ImageJ (NIH, Bethesda) by measuring integrated density in five random fields per section.

### 10× single-cell sequencing

Following the manufacturer's guidelines and a prior study (16), liver tissue fragments were processed through dissociation, filtration, centrifugation, resuspension, and incubation with an erythrocyte lysis buffer. Dead cells were then removed using the Miltenyi® Dead Cell Removal Kit (MACS, #130-090-101; Miltenyi Biotech). Viable cells were suspended in PBS with 0.04% bovine serum albumin, and viability was confirmed via trypan blue exclusion. The cell concentration was adjusted to 700–1200 cells/μl. The single-cell suspension was processed on the 10× Chromium platform, with complementary DNA (cDNA) amplification and library construction being performed using LC-Bio Technology Co., Ltd. Sequencing was conducted on the Illumina NovaSeq 6000 system (Illumina Inc.,).

### Hepatic primary macrophage isolation and flow cytometry

Flow cytometry was used to detect the infiltration level of mouse liver macrophages and isolate mouse liver primary macrophages, as described in our previous study (15). Briefly, anti-mouse CD16/32 antibody (#156603; BioLegend) was used to prevent nonspecific binding for 10 min, followed by staining with antibodies against the cell surface markers CD11b (#101205; BioLegend) and F4/80 (#111603; BioLegend) for 30 min at 4°C.

### THP-1 macrophage polarization and immunofluorescence

The THP-1 cell line, obtained from the Chinese Academy of Sciences, was tested for mycoplasma before use. Cells were cultured in RPMI 1640 with 10% fetal bovine serum (Pricella) and 1% penicillin-streptomycin (Solarbio) at 37°C, 5% CO_2_. Differentiation into M0 macrophages was induced by using 10 ng/ml phorbol-12-myristate-13-acetate (Beyotime) for 24 h; the obtained macrophages were rested for 24 h in a phorbol-12-myristate-13-acetate-free medium. M1 polarization was induced by using 100 ng/ml lipopolysaccharide (#L2630, Sigma-Aldrich) plus 20 ng/ml recombinant human IFN-γ (Animal-Free, #CM081, Sino Biological) for 24 h. M2 polarization was induced by using 20 ng/ml recombinant human IL-4 (Animal-Free, #CM006, Sino Biological) plus 20 ng/ml recombinant human IL-13 (#PHC0136, Gibco Inc.) for 24 h. Successful polarization was confirmed by quantitative real-time PCR (qRT-PCR) for M1 markers (*IL-1β*, *TNF-α*) and M2 markers (*ARG1*, *MRC1*, *IL10*).

For immunofluorescence, cells on coverslips were fixed with 4% paraformaldehyde (15 min), permeabilized with 0.2% Triton X-100, and blocked with 5% normal goat serum. Primary antibodies (same as for IHC: FRMD4B #bs-8236R, Bioss; PTK2B/PYK2 #bsm-52553, Bioss; CPM #DF3899, Affinity; SPTLC2 #DF12231, Affinity; EPB41L2 #15437-1-AP, Proteintech) were incubated overnight at 4°C, after which an Alexa Fluor 488-conjugated goat anti-rabbit secondary antibody (#A11008, 1:500, Invitrogen) was incubated for 1 h at room temperature. Nuclei were counterstained with DAPI. Images were acquired using a confocal microscope (Zeiss LSM880), and fluorescence intensity was quantified using ImageJ.

### Quantitative real-time PCR

Total RNA was isolated from tissues and cells and then reverse-transcribed and amplified, as described in our previous study (15). The mRNA levels of all genes were normalized to those of *β-actin*. The primer sequences are listed in Supplemental Table S1.

### Statistical analysis

Data are expressed as the mean ± standard error. Normality was assessed using the Shapiro–Wilk test. For normally distributed data, statistical significance was calculated using the unpaired two-tailed Student’s *t* test; for non-normally distributed data, the Wilcoxon rank-sum test was applied. ROC analysis was performed using the pROC package in R (version 4.3.2), and AUC values were computed with 95% confidence intervals. All statistical tests were two-tailed, with *P* < 0.05 considered statistically significant.

## Results

### scRNA-seq analysis and cell subpopulation annotation in ScRNA-seq data

**Fig. 1:**
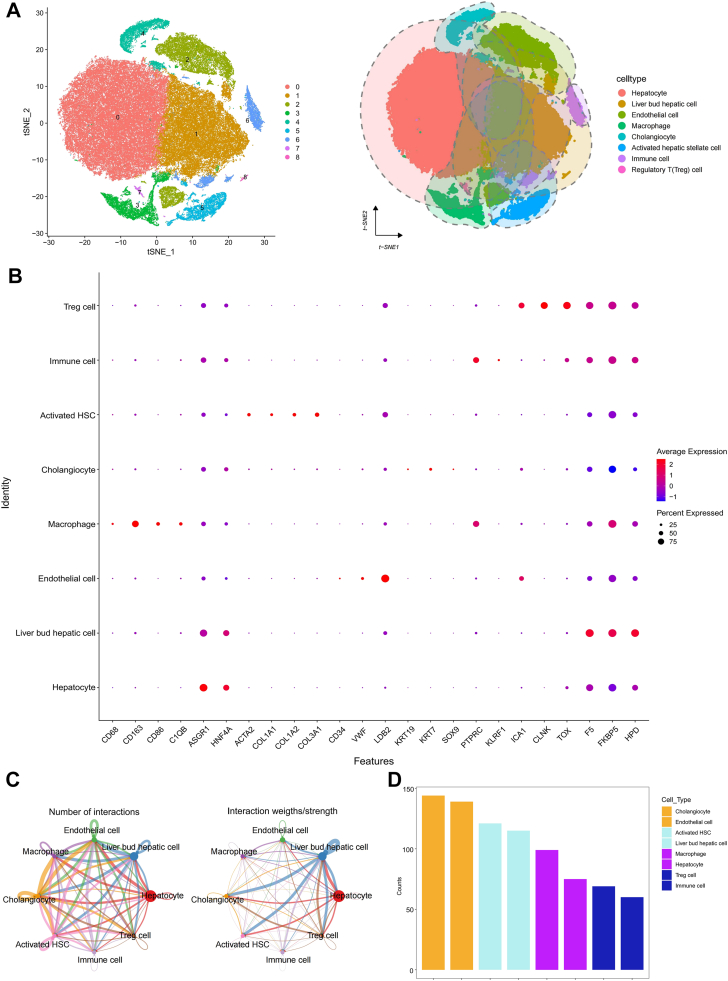
Single-cell-level analysis and cell subpopulation annotation in liver single-cell RNA sequencing data from patients with metabolic dysfunction-associated steatohepatitis (MASH). A: T-distributed stochastic neighbor-embedding diagram of a single-cell sample. Different colors represent specific populations of immune cells. B: Bubble chart presenting the expression of canonical marker genes used to annotate the eight major cell types. Dot size represents the percentage of cells expressing the gene, and color intensity represents the average expression level. C: Circle plot depicting the inferred intercellular communication network between different cell types, with edge width proportional to communication probability. D: Bar plot comparing the total number of outgoing interactions from each cell type.

Following rigorous quality control, we analyzed a total of 100,119 high-quality single cells from the GSE212837 dataset (Supplemental Fig. S1A, B). After normalization and batch effect correction using Harmony (Supplemental Fig. S1C, D), we selected the optimal number of principal components based on the elbow plot (Supplemental Fig. S1E). We performed dimensionality reduction and clustering, which revealed nine distinct cell clusters at a resolution of 0.2 using *t*-SNE (Fig. 1A). Based on well-established marker genes, these clusters were annotated as eight major cell types: hepatocytes, liver bud hepatic cells, endothelial cells, macrophages, cholangiocytes, activated hepatic stellate cells, immune cells, and regulatory T (Treg) cells (Fig. 1B). Using the “FindAllMarkers” function, we identified unique marker genes for each cell subtype from the single-cell data (Supplemental Table S2). Ligand–receptor interaction analysis via CellChat further uncovered extensive and specific communication networks between these cell types, particularly highlighting cholangiocytes and endothelial cells as potential interaction hubs (Fig. 1C, D).

### Screening of macrophage-specific differentially expressed genes and prediction model construction

**Fig. 2:**
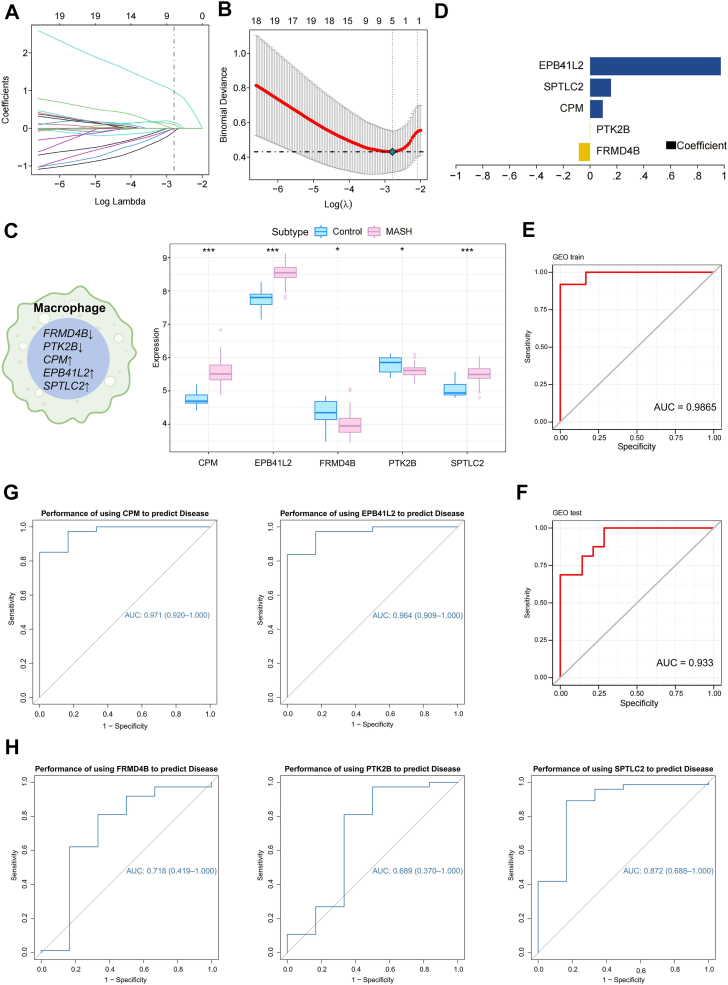
Identification of macrophage-related differentially expressed genes (Mϕ-DEGs) and construction of a diagnostic model for metabolic dysfunction-associated steatohepatitis (MASH). A: Macrophage marker genes screened for features using Lasso regression (avg log_2_FC > 1 and *p*_adj < 0.05). B: Ten-fold cross-validation for tuning parameter (λ) selection in the Lasso model. The *dotted vertical* lines indicate the optimal λ value (lambda.min). C: Expression levels of the five Mϕ-DEGs in the bulk RNA-seq dataset GSE164760. D: formula for calculating the MASH risk score based on the five Mϕ-DEGs. E and F: Receiver operating characteristic (ROC) curves demonstrating the diagnostic performance of the model in the (E) training (GSE164760) and (F) independent validation (GSE126848) sets. G and H: ROC curves presenting the predictive power of each Mϕ-DEG.

Given the central role of macrophages in MASH pathogenesis (5), we sought to identify macrophage-specific biomarker genes. We used the GSE164760 and GSE126848 datasets for training and validation, respectively. We applied Lasso regression to identify key Mϕ-DEGs (Fig. 2A) and selected the optimal regularization parameter using cross-validation, minimizing prediction error (Fig. 2B). Lasso regression identified five feature genes: FERM domain containing 4B (*FRMD4B*), protein tyrosine kinase 2 β (*PTK2B*), carboxypeptidase M (*CPM*), serine palmitoyltransferase long chain base subunit 2 (*SPTLC2*), and erythropoietin-producing hepatocellular carcinoma receptor B41-like 2 (*EPB41L2*). *FRMD4B* and *PTK2B* expression levels were significantly lower in MASH livers than in control livers, whereas *CPM*, *EPB41L2*, and *SPTLC2* expression levels were significantly higher (Fig. 2C). We subsequently constructed a MASH prediction model incorporating these genes as follows: RiskScore = *FRMD4B* × (−0.0834051104524685) + *PTK2B* × (−0.00117237954685) + *CPM* × 0.093845047170987 + *SPTLC2* × 0.155417260889053 + *EPB41L2* × 0.974527032809338 (Fig. 2D). The model demonstrated high diagnostic accuracy, with an AUC of 0.9865 in the training set and 0.933 in the independent validation set (GSE126848) (Fig. 2E, F). Notably, *CPM*, *SPTLC2*, and *EPB41L2* alone also exhibited high predictive power, with AUC values of 0.971, 0.872, and 0.964, respectively (Fig. 2G, H).

### Analysis of the expression of Mϕ-DEG in various cell populations and macrophage subpopulations within the livers of patients with MASH

**Fig. 3:**
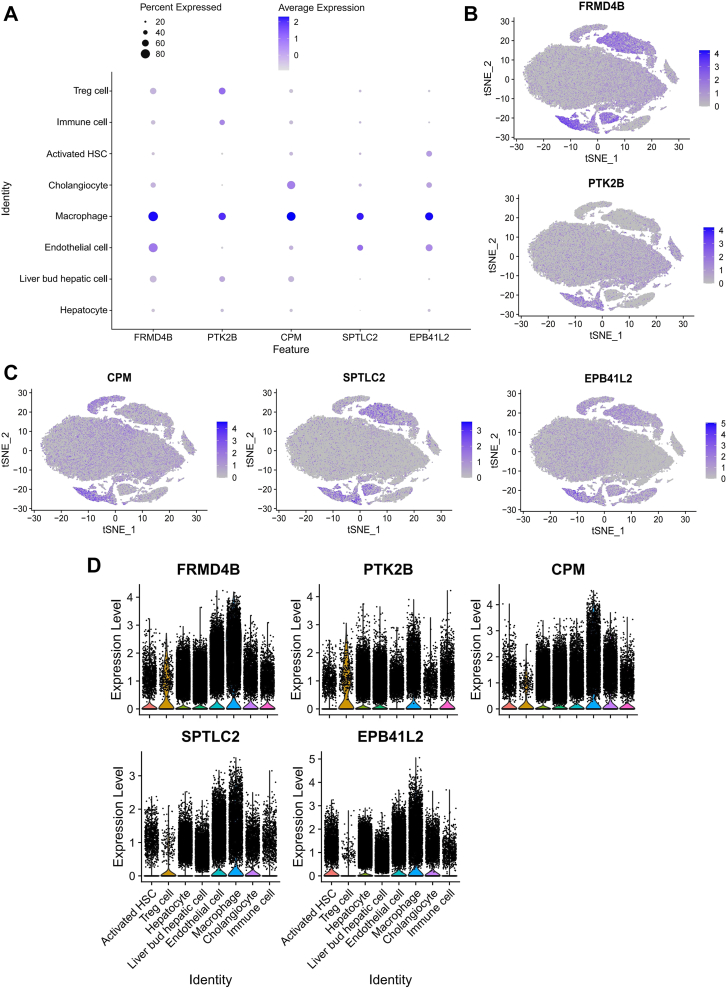
Cellular specificity of macrophage-related differentially expressed genes (Mϕ-DEGs) expression in human metabolic dysfunction-associated steatohepatitis (MASH) livers. A: Bubble chart displaying the expression patterns of the five Mϕ-DEGs among the eight major hepatic cell types. Color intensity represents the mean expression level, and dot size indicates the percentage of expressing cells within each cluster. B–D: Feature plots (B, C) and violin plots (D) quantifying the expression levels of the five Mϕ-DEG across different cell types.

We next examined the cellular specificity of the five Mϕ-DEGs within the MASH liver landscape by analyzing their expression profiles across major hepatic cell types using scRNA-seq data. Notably, all five genes were enriched in macrophages, with lower expression levels in hepatocytes (Fig. 3A–D).

**Fig. 4:**
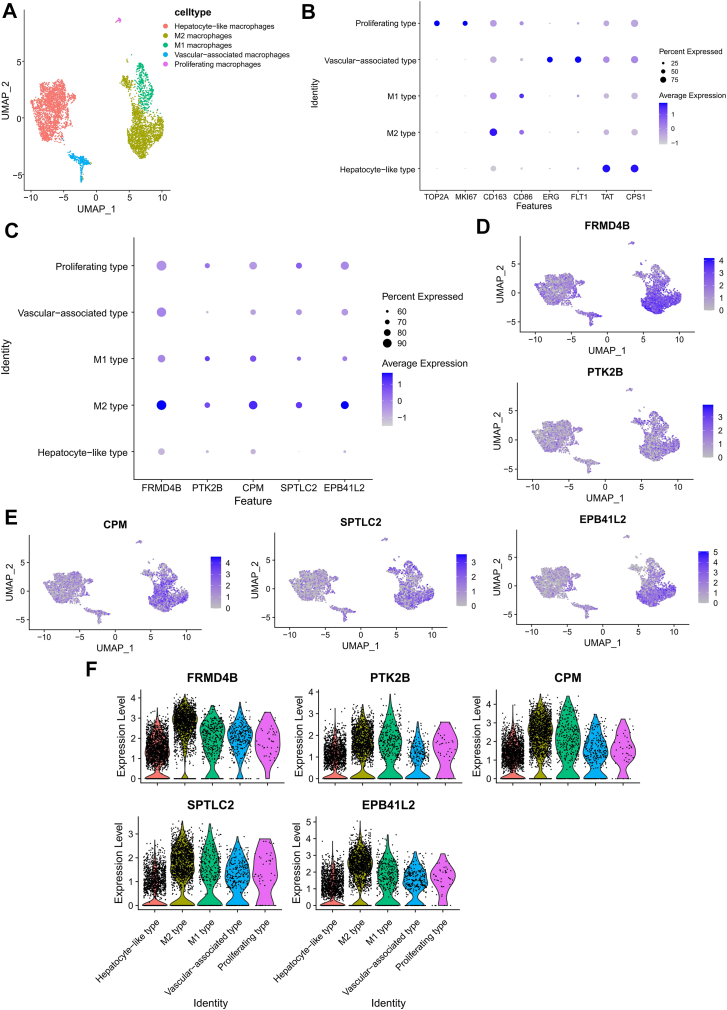
Expression of macrophage-related differentially expressed genes (Mϕ-DEGs) across macrophage subpopulations in human metabolic dysfunction-associated steatohepatitis (MASH) livers. A: T-distributed stochastic neighbor embedding plot showing the annotated macrophage subpopulations. B: Expression of canonical marker genes for the macrophage subpopulations. C: Expression patterns of the five Mϕ-DEGs across macrophage subpopulations. Color intensity represents the mean expression level, and dot size indicates the percentage of expressing cells within each cluster. D–F: Expression distribution of five Mϕ-DEGs among macrophage subpopulations.

We further analyzed macrophage heterogeneity and observed distinct subpopulations, including M1, M2, vascular-associated, proliferating, and hepatocyte-like macrophages (Fig. 4A, B). Analysis of Mϕ-DEG expression across these subsets revealed a striking and consistent enrichment within M2 macrophages, with a moderate expression being detected in proliferating macrophages (Fig. 4C–F).

### Validation of Mϕ-DEG expression in human and murine MASH models

**Fig. 5:**
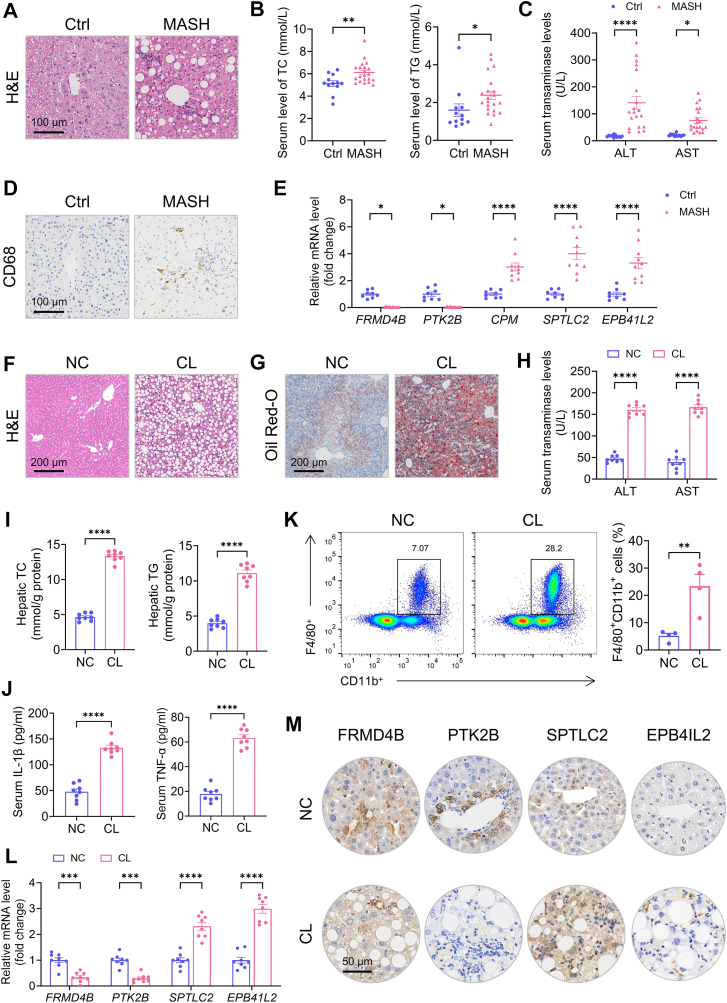
Validation of macrophage-related differentially expressed genes (Mϕ-DEGs) expression and metabolic dysfunction-associated steatohepatitis (MASH) phenotype in human patients and mouse models. A: Representative H&E-stained human liver sections from control (adjacent to hepatic hemangioma) and MASH patients. B and C: Serum levels of lipids (TC, TG) and liver enzymes (ALT, AST) in human controls (n = 12) and MASH patients (n = 20). D: Representative CD68 immunohistochemistry in human liver sections. E: qRT-PCR analysis of the five Mϕ-DEG mRNA levels in human liver tissues (Ctrl n = 8, MASH n = 10). F and G: Representative H&E (F) and Oil Red O (G) staining of mouse livers from NC and CL diet-fed mice. H: Serum ALT and AST levels in mice (n = 8 per group). I: Hepatic TC and TG contents (n = 8 per group). J: Serum IL-1β and TNF-α levels (n = 8 per group). K: Flow cytometry analysis of hepatic F4/80^+^CD11b^+^ macrophage infiltration (n = 4 per group). L: qRT-PCR analysis of the five Mϕ-DEGs in mouse livers (n = 8 per group). M: Immunohistochemistry of FRMD4B, PTK2B, SPTLC2, and EPB41L2 in mouse livers (CPM protein undetectable). Data are presented as means ± SEM; ∗*P* < 0.05, ∗∗*P* < 0.01, ∗∗∗*P* < 0.001, ∗∗∗∗*P* < 0.0001 (unpaired *t* test). n.s., not significant.

The expression and clinical relevance of these five Mϕ-DEGs were validated in human and murine MASH samples. Histological analysis via H&E staining revealed that inflammatory cell infiltration, lipid deposition, and hepatocyte enlargement were significantly increased in the liver tissues of patients with MASH compared to normal paratumoral tissues (Fig. 5A). Serum levels of total cholesterol and triglycerides were significantly elevated in MASH patients (Fig. 5B), as were alanine aminotransferase and aspartate aminotransferase (Fig. 5C). CD68-positive cell infiltration was markedly increased in MASH livers (Fig. 5D). qRT-PCR confirmed significant downregulation of *FRMD4B* and *PTK2B* mRNA and upregulation of *CPM*, *SPTLC2*, and *EPB41L2* in human MASH tissues (Fig. 5E).

Consistent with human data, mice fed a CL diet developed typical MASH phenotypes: H&E staining showed increased inflammation and hepatocyte ballooning (Fig. 5F), and Oil Red O staining revealed enhanced lipid accumulation (Fig. 5G). Serum ALT and AST levels were elevated (Fig. 5H), as were hepatic TC and TG contents (Fig. 5I). Serum pro-inflammatory cytokines IL-1β and TNF-α were also increased (Fig. 5J). Flow cytometry demonstrated increased hepatic F4/80^+^CD11b^+^ macrophage infiltration in CL-fed mice (Fig. 5K). qRT-PCR confirmed significant downregulation of *Frmd4b* and *Ptk2b* mRNA and upregulation of *Sptlc2* and *Epb41l2* in mouse livers, with Cpm undetectable (Fig. 5L). immunohistochemistry (IHC) analysis of mouse livers showed similar protein-level changes for *Frmd4b*, *Ptk2b*, *Sptlc2*, and *Epb41l2*, whereas Cpm protein was undetectable (Fig. 5M).

### Single-cell expression profiling of Mϕ-DEGs in mouse livers

**Fig. 6:**
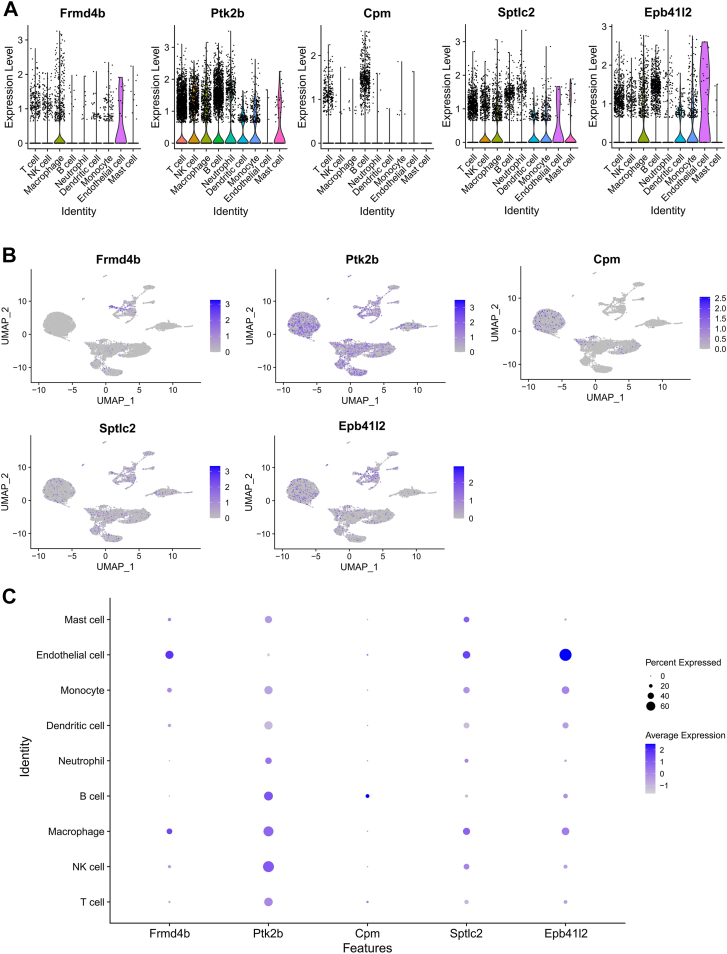
Single-cell expression profiling of macrophage-related differentially expressed genes (Mϕ-DEGs) in murine metabolic dysfunction-associated steatohepatitis (MASH) livers and validation in vitro. A: Average expression (color scale) and percentage of cells expressing (dot size) each Mϕ-DEG across major immune cell types in mouse livers (NC, n = 1; C,L n = 1). B: Expression and distribution of individual Mϕ-DEGs across cells in the t-distributed stochastic neighbor embedding. C: Expression levels of each Mϕ-DEG in different cell populations from mice fed a normal chow (NC) or high-cholesterol and high-fat (CL) diet.

We performed 10× single-cell transcriptome sequencing on liver samples from NC- and CL-fed mice. Figure 6A shows a dot plot of the average expression (color) and percentage of expressing cells (dot size) for *Frmd4b*, *Ptk2b*, *Cpm*, *Sptlc2*, and *Epb41l2* across major hepatic cell types. Figure 6B presents UMAP plots visualizing the distribution of each gene’s expression within the single-cell landscape. Figure 6C displays a bubble plot of gene expression across nine annotated cell populations (T cells, NK cells, macrophages, B cells, neutrophils, dendritic cells, monocytes, endothelial cells, and mast cells). *Frmd4b* and *Cpm* showed relatively low expression in mouse liver, whereas *Ptk2b*, *Sptlc2*, and *Epb41l2* were highly expressed. Except for *Cpm*, the other four genes were highly expressed in macrophages.

### Expression of Mϕ-DEGs in polarized THP-1 macrophages and correlation with M2-related genes

**Fig. 7:**
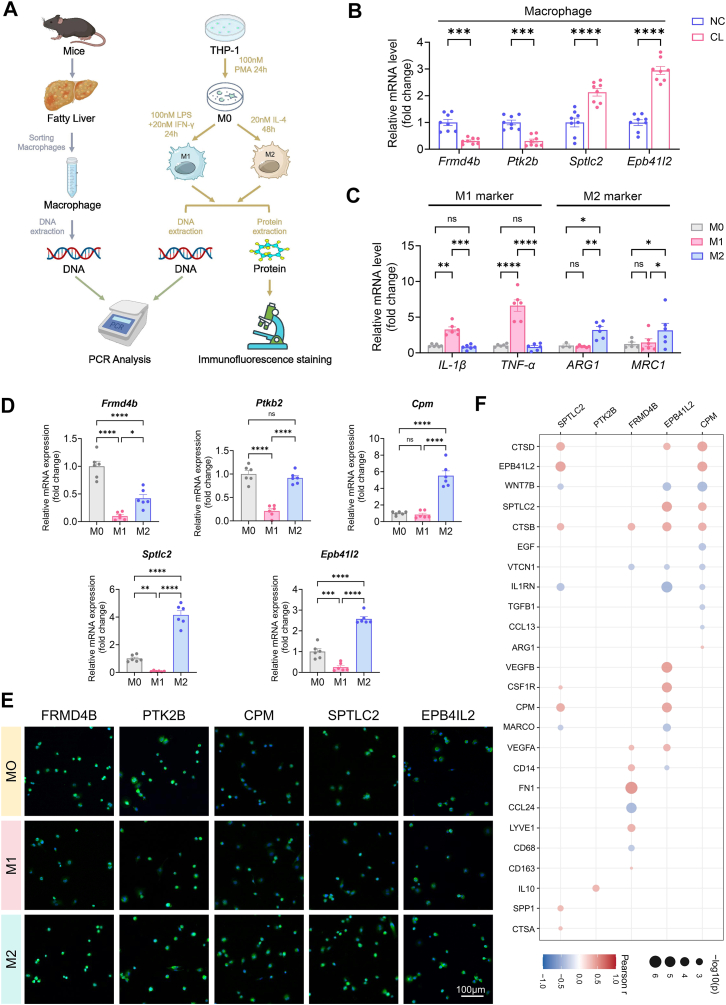
Validation of macrophage-related differentially expressed genes (Mϕ-DEGs) expression in primary mouse hepatocytes and polarized THP-1 macrophages, and correlation with M2-related genes. A: Schematic workflow for mouse hepatocyte isolation and THP-1 polarization experiments. B: qRT-PCR analysis of *Frmd4b*, *Ptk2b*, *Sptlc2*, and *Epb41l2* in primary mouse hepatocytes from NC and CL diet-fed mice. Cpm was undetectable. C: qRT-PCR analysis of M1 markers (*IL-1β*, *TNF-α*) and M2 markers (*ARG1*, *MRC1*) in polarized THP-1 cells. D: qRT-PCR analysis of five Mϕ-DEG in M0, M1, and M2 THP-1 cells. E: Representative immunofluorescence images of the five proteins (green) in M0, M1, M2 cells; nuclei are stained with DAPI (blue). F: Bubble plot showing Pearson correlation coefficients between the five Mϕ-DEGs and M2-related genes in the GSE164760 dataset. Bubble color indicates correlation direction (red positive, blue negative), and bubble size represents the absolute correlation coefficient. Data mean ± SEM. (B) n = 8 per group, (C, D) n = 6 per group. ∗*P* < 0.05, ∗∗*P* < 0.01, ∗∗∗*P* < 0.001, ∗∗∗∗*P* < 0.0001 (unpaired *t* test). n.s., not significant.

We characterized the five Mϕ-DEGs in polarized THP-1 macrophages. Figure 7A illustrates the experimental workflow for macrophage sorting and polarization. In primary mouse hepatocytes isolated from NC and CL diet-fed mice, qRT-PCR detected *Frmd4b*, *Ptk2b*, *Sptlc2*, and *Epb41l2* expression, while *Cpm* was undetectable (Fig. 7B).

THP-1 cells were polarized into M0, M1, and M2 subtypes. qRT-PCR showed upregulation of M1 markers (*IL-1β*, *TNF-α*) in M1 cells and M2 markers (*ARG1*, *MRC1*) in M2 cells (Fig. 7C). qRT-PCR of the five Mϕ-DEGs showed that *FRMD4B* and *PTK2B* mRNA levels were higher in M0 cells and lower in M1 and M2 cells, while *CPM*, *SPTLC2*, and *EPB41L2* mRNA levels were higher in M2 cells than in M0 or M1 cells (Fig. 7D). Immunofluorescence staining for the five proteins (green) in M0, M1, and M2 cells (nuclei blue) showed that FRMD4B and PTK2B signals were stronger in M0 cells and weaker in M1/M2, whereas CPM, SPTLC2, and EPB41L2 signals were stronger in M2 cells (Fig. 7E).

To evaluate the association between Mϕ-DEGs and M2 polarization at the population level, Pearson correlation analysis was performed using the GSE164760 bulk transcriptome dataset. As shown in Fig. 7F, *CPM* was positively correlated with *ARG1*, and *PTK2B* was positively correlated with *IL-10*. *EPB41L2* and *SPTLC2* were positively correlated with *CTSB*, *CTSD*, and *CSF1R*, while *FRMD4B* was positively correlated with *CD163* and *FN1* but negatively correlated with *CD68*.

### Mϕ-DEGs are correlated with immune microenvironment remodeling

**Fig. 8:**
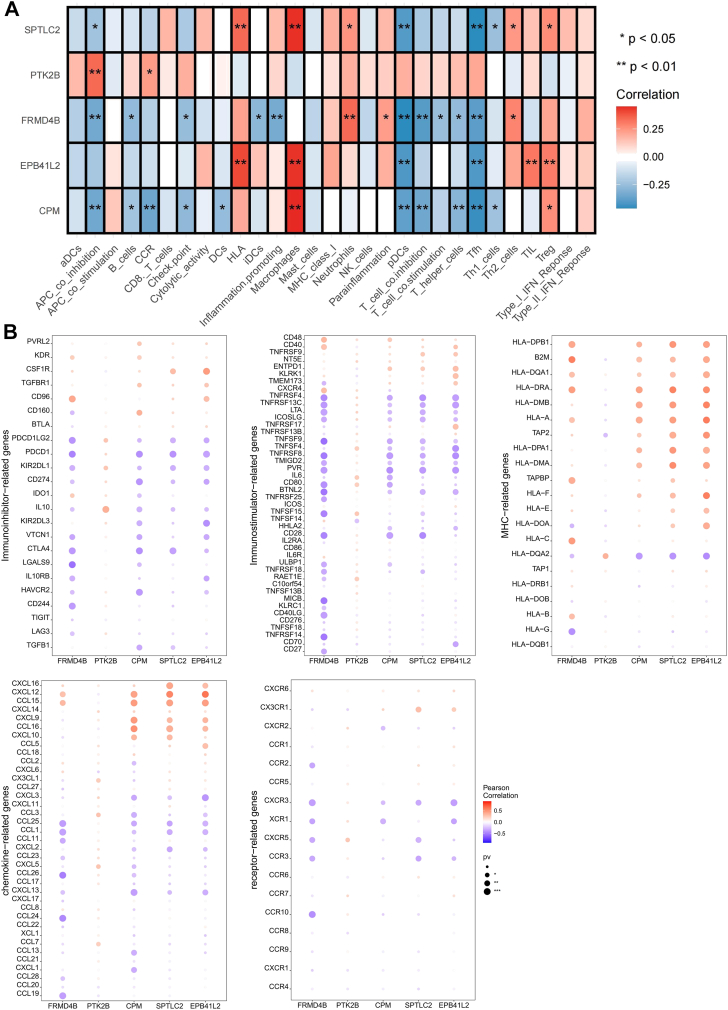
Correlation between macrophage-related differentially expressed genes (Mϕ-DEGs) and the immune microenvironment in metabolic dysfunction-associated steatohepatitis (MASH). A: Heatmap of Pearson correlation coefficients between the expression of the five Mϕ-DEGs and the relative abundance of various immune cell types, as estimated by ssGSEA. Red indicates positive correlation; blue indicates negative correlation. B: Bubble plots depicting the correlation between the five Mϕ-DEGs and the expression of key immunomodulators, including immunoinhibitors, immunostimulators, major histocompatibility complex (MHC) molecules, chemokines, and chemokine receptors. Bubble color and size represent the correlation coefficient (r) and its statistical significance (-log10(*P*-value)), respectively.

The immunoregulatory roles of the Mϕ-DEGs were explored by immune infiltration analysis using CIBERSORT. The MASH microenvironment was characterized by increased infiltration of macrophages, Th2, and Treg cells, but decreased T follicular helper (Tfh) cells (Supplemental Fig. S3A–C). Correlation analysis revealed that all Mϕ-DEGs except for *PTK2B* were negatively correlated with Tfh cell infiltration. Furthermore, *FRMD4B* expression was positively correlated with Th2 cells, whereas *CPM*, *SPTLC2*, and *EPB41L2* exhibited strong positive correlations with macrophages and Treg cells (Fig. 8A). These genes were also significantly associated with the expression of various immunomodulators, including cytokines, chemokines, and immune checkpoint molecules (Fig. 8B), suggesting their pivotal role in shaping the immunosuppressive microenvironment in MASH.

### Functional enrichment and regulatory network of Mϕ-DEGs

We elucidated the functional pathways of the five Mϕ-DEGs by performing GSEA and GSVA. The results indicated distinct pathway enrichments for each gene: *FRMD4B* in adipocytokine, FoxO, and lipoic acid metabolism; *PTK2B* in cytochrome P450-mediated drug metabolism, ferroptosis, and IL-17 signaling; *CPM* in AMPK, fatty acid, and PPAR pathways; *SPTLC2* in fatty acid metabolism, sphingolipid, and TGF-β signaling; and *EPB41L2* in mRNA surveillance, PI3K/Akt, and TGF-β pathways (Supplemental Figs. S4A–E and S5). Notably, all five genes were broadly associated with fatty acid metabolism and androgen response in GSVA (Supplemental Figs. S4F–J and S5). Furthermore, *FRMD4B* correlated with MYC targets V1 and IL2/STAT5 signaling; *PTK2B* with MYC targets V2; *CPM* with heme metabolism; *SPTLC2* with TGF-β and PI3K/AKT/mTOR signaling; and *EPB41L2* with ultraviolet response and TGF-β signaling (Supplemental Figs. S4F–J and S5).

**Fig. 9:**
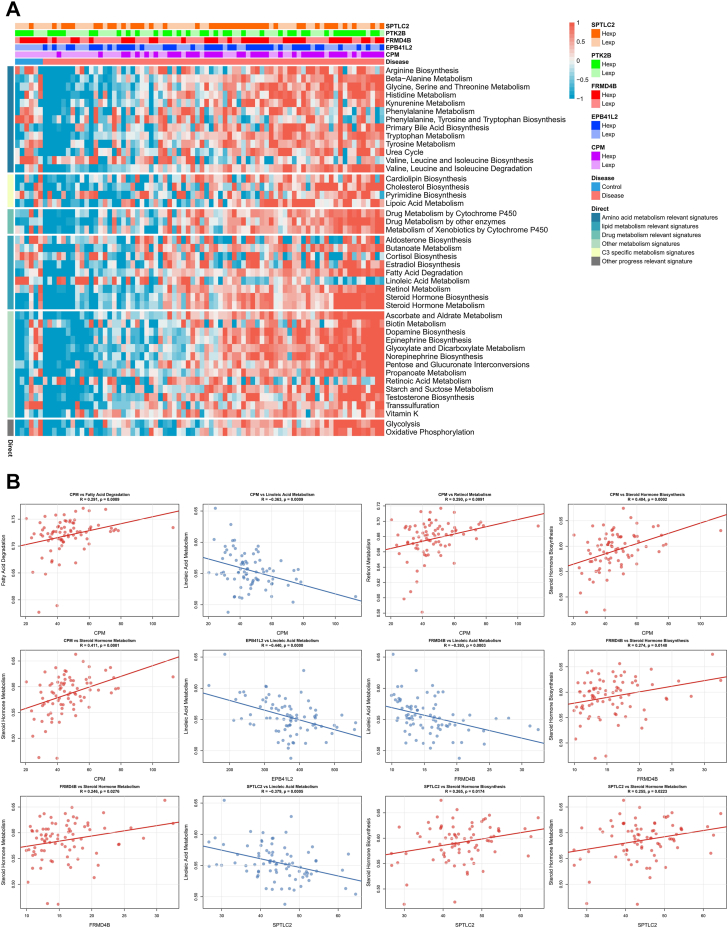
Correlation of macrophage-related differentially expressed genes (Mϕ-DEGs) with lipid metabolic pathways in metabolic dysfunction-associated steatohepatitis (MASH). A: Heatmap of Pearson correlations between each Mϕ-DEG and ssGSEA scores of lipid-related pathways in GSE164760. B: Representative scatter plots. Linear regression lines with 95% CI.

To further explore the link between Mϕ-DEGs and lipid metabolism, we performed an ssGSEA-based pathway activity correlation analysis. Figure 9A shows a heatmap of Pearson correlations between each Mϕ-DEG and selected lipid-related pathways (fatty acid degradation, linoleic acid metabolism, retinol metabolism, steroid hormone biosynthesis, and steroid hormone metabolism). Notably, *CPM*, *SPTLC2*, and *EPB41L2* showed significant positive correlations with steroid hormone biosynthesis and metabolism, whereas *FRMD4B* and *PTK2B* exhibited weaker or negative correlations. Figure 9B provides representative scatter plots illustrating these associations (e.g., *CPM* positively correlated with steroid hormone metabolism, R = 0.411, *P* = 0.0001; *EPB41L2* negatively correlated with linoleic acid metabolism, R = −0.440, *P* < 0.0001). These findings align with the GSEA results and support the involvement of these genes in lipid metabolic reprogramming in MASH.

### Relationship with known MASH genes and regulatory network

We next examined the relationship between the Mϕ-DEGs and known MASH pathogenesis genes using GeneCards. Among the top 20 candidates, glutamate pyruvate transaminase (*GPT*), keratin polypeptides 18 (*KRT18*), and *IL6* were significantly downregulated, whereas *HSD17B13* was upregulated in disease samples (Supplemental Fig. S6A). Correlation analysis revealed that *CPM*, *EPB41L2*, and *SPTLC2* expression were positively associated with *HSD17B13* but negatively correlated with *GPT*. *FRMD4B* was inversely correlated with *KRT18* (r = −0.257, *P* = 0.021), and *PTK2B* was positively correlated with *IL6* (r = 0.242, *P* = 0.031) (Supplemental Fig. S6B).

Finally, we analyzed transcriptional control of the five Mϕ-DEGs. TF enrichment using recovery curves identified cisbp__M2259 as the most significantly enriched motif (NES = 6.85). We constructed a comprehensive TF–miRNA–mRNA regulatory network to illustrate potential upstream mechanisms governing these genes (Supplemental Fig. S7A, B).

### Mendelian randomization analysis implicates causal roles of Mϕ-DEGs in MASH

Potential causal relationships were investigated using two-sample Mendelian randomization (MR) analysis. As presented in Figure S8, the primary IVW method identified *CPM* as a significant causal protective factor for MASH (odds ratio = 0.375, 95% confidence interval: 0.251–0.560, IVW *P* = 0.014). For *EPB41L2*, although the IVW estimate was nonsignificant (*P* > 0.05), the weighted median method yielded a nominally significant protective effect (odds ratio = 0.601, 95% confidence interval: 0.646–0.777, weighted median *P* = 0.048), providing suggestive evidence for a causal role. Analyses for *PTK2B*, *FRMD4B*, and *SPTLC2* did not yield statistically significant evidence for a causal effect across primary or sensitivity methods (all *P* > 0.05).

## Discussion

The growing global health burden of MASLD underscores the urgent need to understand its cellular mechanisms and identify reliable biomarkers (1, 2). Although hepatic macrophages are central to this process (4, 14), their specific molecular drivers remain elusive. In the present study, our integrated multi-omics approach identified five macrophage-enriched genes (*FRMD4B, PTK2B, CPM, SPTLC2*, and *EPB41L2*) as robust diagnostic biomarkers (AUC = 0.9865) that are critically involved in reshaping the MASH microenvironment, with pronounced enrichment in M2-polarized macrophages.

Among the five identified genes, *CPM*, *SPTLC2*, and *EPB41L2* constitute a compelling triad of putative compensatory regulators. Despite their significant upregulation in MASH, MR analysis supported a genetically protective role for *CPM*, with a similar trend observed for *EPB41L2* in sensitivity analyses. This apparent paradox suggests that their elevated expression may represent a compensatory host response to counteract disease progression. This view is further supported by their shared positive correlation with the protective gene *HSD17B13* (17) and negative correlation with the hepatic injury marker *GPT*. The protein-level upregulation of these genes in MASH livers and their enrichment in M2-polarized THP-1 macrophages further consolidated their association with M2-driven immunometabolic remodeling. Consistent with these findings, bulk transcriptome correlation analysis further confirmed that *CPM*, *SPTLC2*, and *EPB41L2* were positively associated with M2-related genes (e.g., *ARG1*, *CTSB*, *CSF1R*) in an independent cohort.

Functionally, *CPM*, which is highly expressed in lipid-laden macrophages (18) and enriched in AMPK/PPAR pathways, aligns with its role in lipid metabolism. *SPTLC2*, the core component of serine palmitoyltransferase, regulates sphingolipid metabolism and TGF-β signaling, consistent with its reported role in promoting cholesterol excretion (19). *EPB41L2*, originally identified as an erythrocyte membrane skeleton protein (20) and a tumor suppressor (21), impairs phagocytosis and enhances VEGF-A secretion in M2 macrophages via PI3K/AKT (22); our data also show its participation in TGF-β signaling.

The other two genes, *FRMD4B* and *PTK2B*, were downregulated in MASH. *FRMD4B* promotes the remodeling of cell connections and the dynamic changes of the cytoskeleton (23, 24) and is related to insulin signaling and liver glucose-lipid homeostasis (25, 26, 27). Our results indicated that *FRMD4B* participates in the pathogenesis of MASH by regulating pathways such as adipocyte factor (28), FoxO (29), and thiamine metabolism (30). Moreover, *FRMD4B* exhibits a significant negative correlation with the liver cell injury marker *KRT18* (31), suggesting that its downregulation may exacerbate epithelial damage. *PTK2B*, a member of the focal adhesion kinase family known to regulate macrophage migration, polarization, and inflammatory responses (32, 33, 34), was consistently downregulated. This finding is consistent with reports that the inhibition of *PTK2B* alleviates MASLD and MASH-related HCC (35). Its unique positive correlation with *IL6* suggests a complex, context-dependent pro-inflammatory facet, potentially mediated through its role in macrophage behavior. However, the absence of significant MR evidence for both genes indicates that their expression changes are more likely consequences, rather than primary drivers, of MASH. Conversely, *FRMD4B* and *PTK2B* showed negative or weak correlations with these M2-related genes.

Beyond individual genes, we observed a remodeled immune landscape characterized by increased Th2/Treg cells and decreased Tfh cells. The strong negative correlation between most Mϕ-DEGs and Tfh cells posits a novel link between macrophage-specific signals and the suppression of adaptive humoral immunity in MASH (36, 37), which is a process known to be influenced by metabolic cues like leptin and to interact with macrophage polarization (38, 39, 40). Therefore, these results warrant future investigation.

This study has several limitations. First, although we validated CPM in human tissues and THP-1 cells, the species-specific absence of CPM in mice limits rodent models for CPM studies (41). Second, an in-depth multi-omics dissection of macrophage heterogeneity was not performed (42). Third, the immune microenvironment associations were correlative; while we provided protein-level validation, direct functional studies (gene perturbation, co-culture, in vivo mechanisms) are needed to establish causality. Additionally, we did not isolate M2 macrophages from MASH livers by FACS for gene-specific protein analysis, which is a research gap that should be addressed in future investigations.

In conclusion, by analyzing the roles of five macrophage-centric genes, our results advance beyond a biomarker signature towards a more refined model of MASH pathogenesis. The compensatory triad of *CPM*, *SPTLC2*, and *EPB41L2* within M2 macrophages appears to form a critical network that actively shapes the immunometabolic landscape, thereby offering promising subtype-specific therapeutic targets for this intractable disease.

## Data availability

The GSE212837, GSE164760, and GSE126848 datasets were obtained from the Gene Expression Omnibus (GEO) databases. The complete R analysis code and processed Seurat objects have been deposited to Figshare (https://doi.org/10.6084/m9.figshare.12345678). Raw and processed 10× single-cell data for mouse livers are available at https://www.ncbi.nlm.nih.gov/bioproject/PRJNA1161452.

## Supplemental data

This article contains supplemental data.

## Conflicts of interest

The authors declare that they have no conflicts of interest with the contents of this article.
