# Supplementary material for: Unveiling novel macrophage-specific biomarkers in MASH through single-cell sequencing for diagnostic modeling
Source: J Lipid Res. 2026 Apr 28;67(6):101048. doi: 10.1016/j.jlr.2026.101048 (PMC13226250; doi:10.1016/j.jlr.2026.101048)
Supplement: Supplementary Figures [file mmc1.pdf]

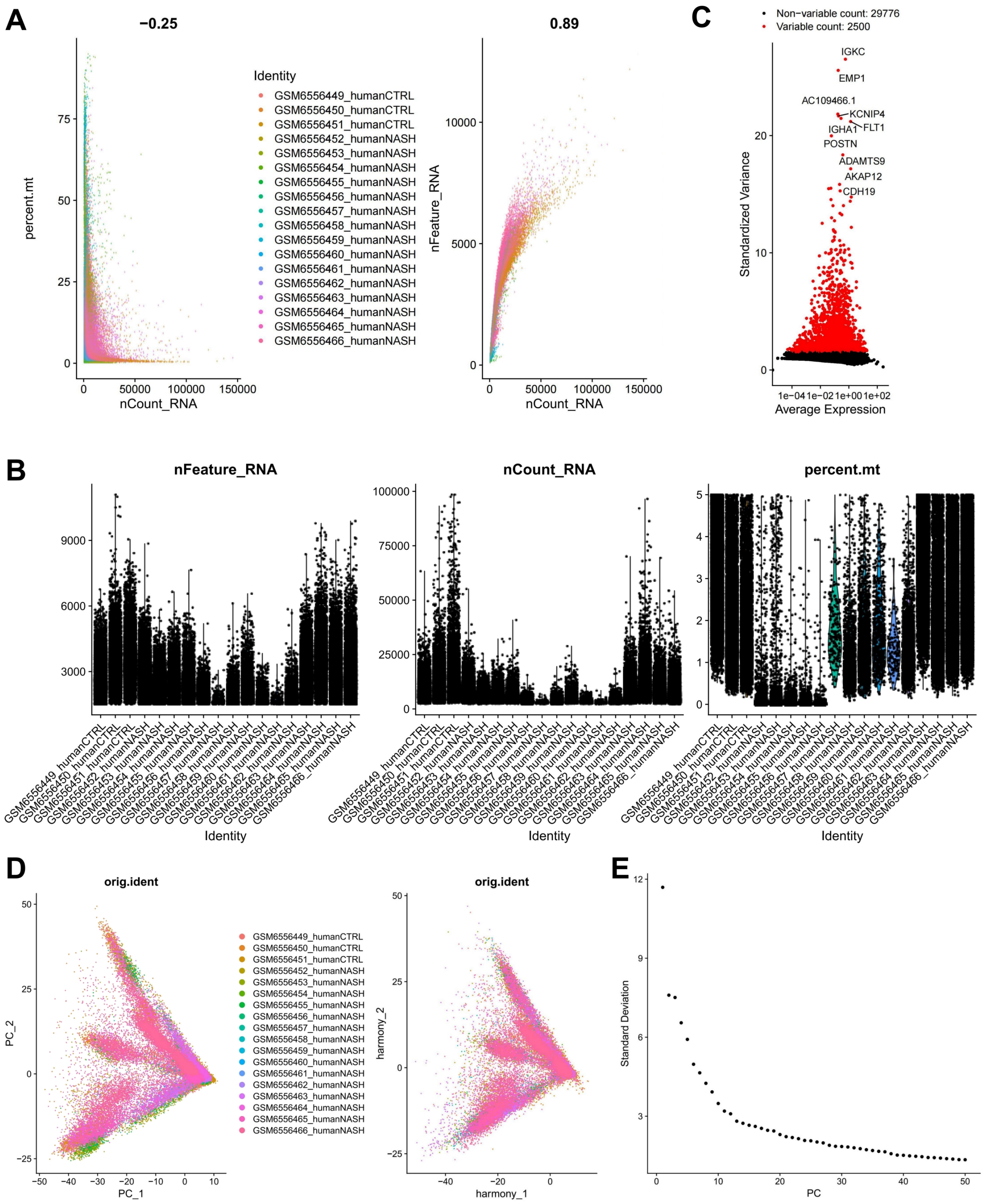


**Fig. S1. Quality control and preprocessing of human MASH scRNA-seq data (GSE212837).** A: Single-cell quality control (nFeature_RNA > 1500, percent.mt < 5, nCount_RNA < 100 000) on the GSE212837 dataset; dots represent cells, whereas colours represent samples. B: Bar plots showing the total cell count, median gene count, and median UMI count per sample after QC filtering. C: Plot representing the standard deviation of genes versus their average expression, highlighting the top ten most variable genes (red). D: Visualization of batch effect correction using PCA (left) and Harmony (right) algorithms. Points represent cells, colored by sample origin. E: Elbow plot used to determine the optimal number of principal components (PCs) for downstream analysis, indicating a cutoff at 10 PCs.

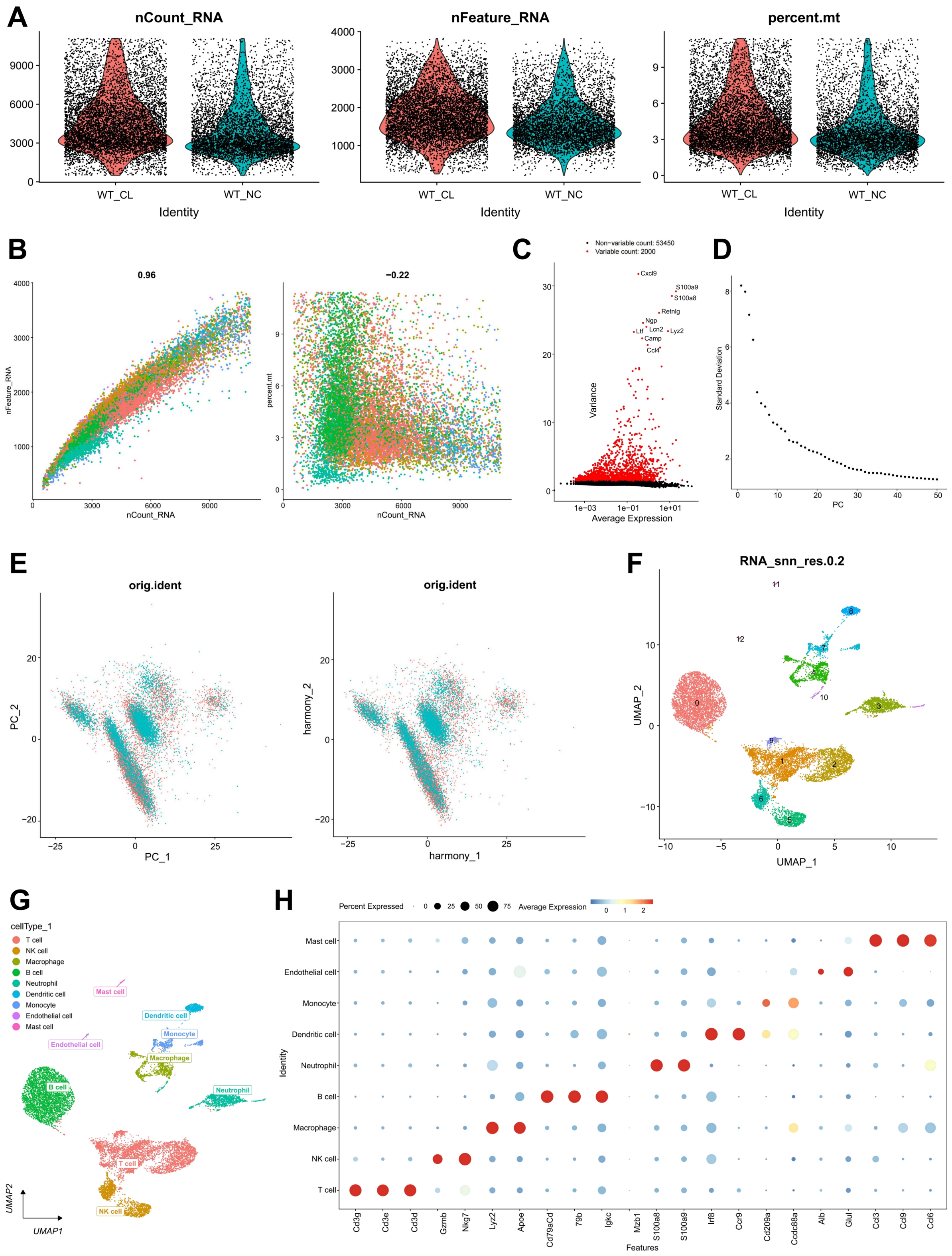


**Fig. S2. scRNA-seq analysis of the murine metabolic dysfunction-associated steatohepatitis (MASH) model.** A: Quality control metrics (cell count, gene count, sequencing depth) for each mouse liver sample; mice were either fed normal chow (NC) or high-cholesterol and high-fat (CL) diet (NC n=1; CL n=1). B: Scatter plots of sequencing depth versus the number of genes detected per cell (left) and versus the percentage of mitochondrial reads (right). C: Plot of the standard deviation of genes versus their average expression, identifying highly variable genes. D: Elbow plot for determining the number of PCs. E: Dimensionality reduction visualization using PCA (left) and

Harmony-corrected UMAP (right), colored by sample. F: *t*-SNE plot colored by unsupervised cell clusters (0-12). G: *t*-SNE plot colored by annotated immune cell types. H: Dot plot (DotPlot) displaying the expression of canonical marker genes for the nine identified immune cell types.

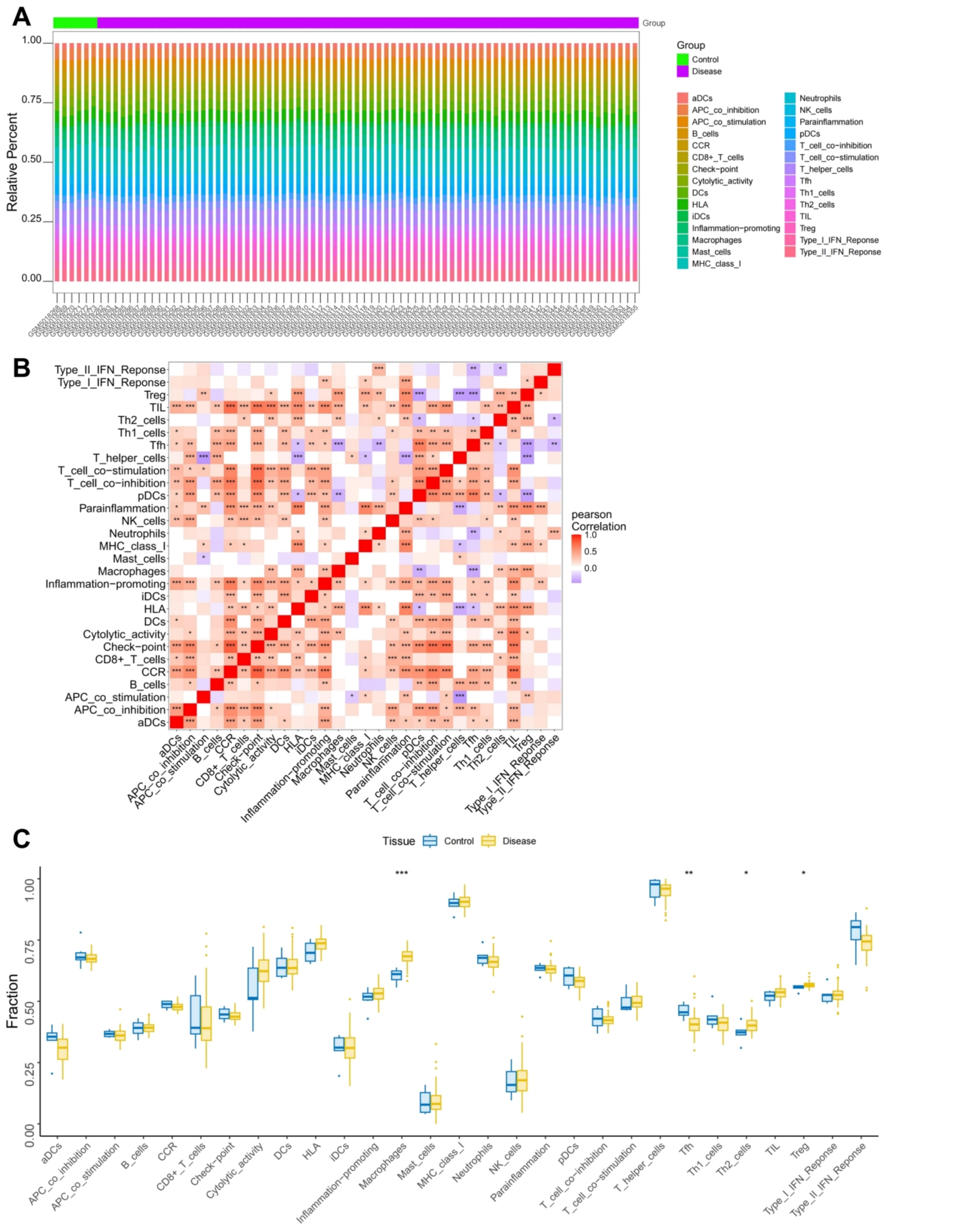


**Fig. S3. Intrahepatic immune infiltration in patients with MASH.** A: Stacked bar plot showing the relative proportion of immune cell subsets across all samples. B: Correlation matrix of immune cell abundances. Purple indicates negative correlation, red indicates positive correlation. C: Violin plots comparing the infiltration levels of immune cells between control (blue) and MASH (yellow) groups. **P* < 0.05, ***P* < 0.01, ****P* < 0.001 (unpaired *t*-test).

# Figure 8

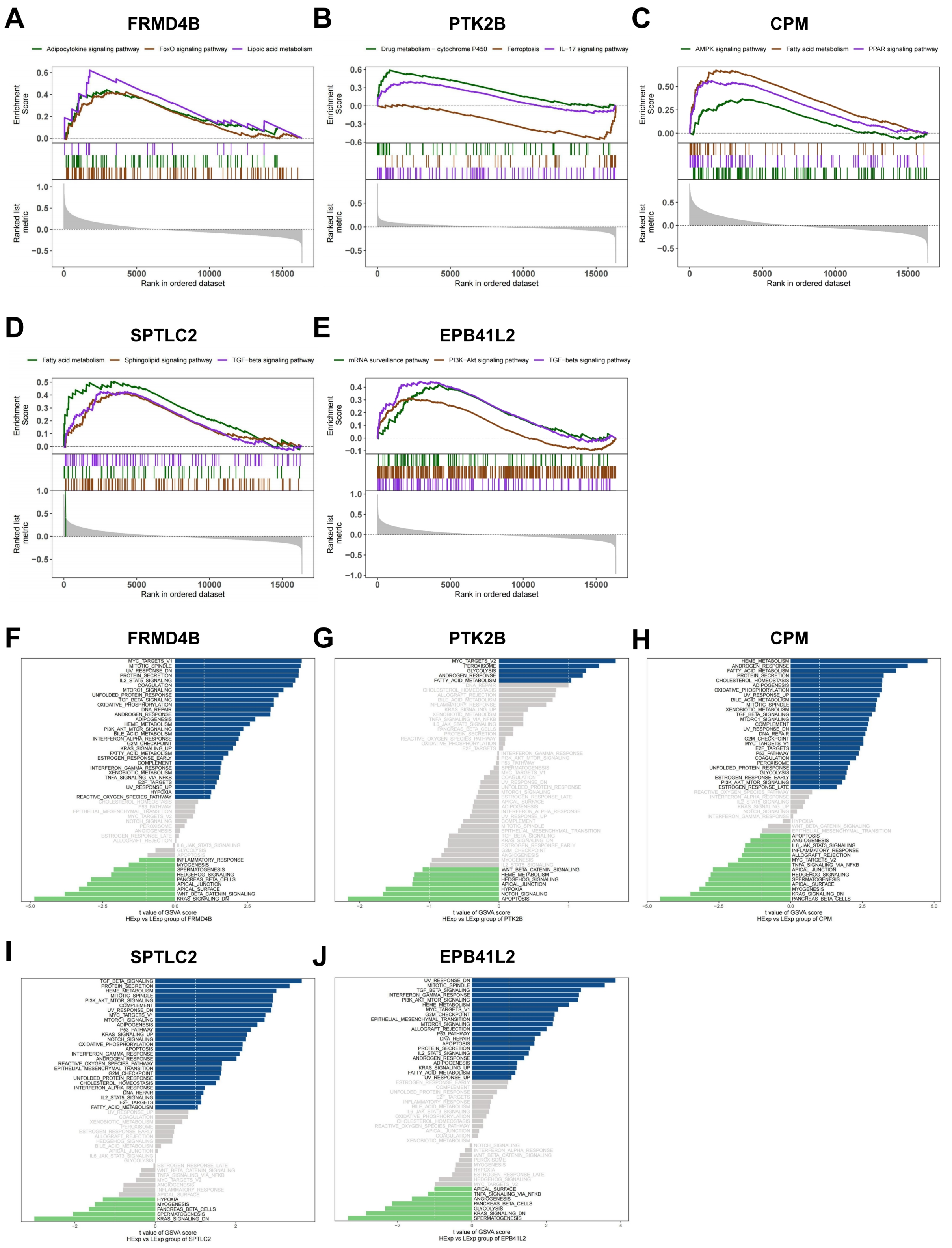


**Fig. S4. Signaling pathway enrichment analysis of macrophage-related differentially expressed genes (Mφ-DEGs) in metabolic dysfunction-associated steatohepatitis (MASH).** A–E: Gene set enrichment analysis plots for each Mφ-DEG, showing the top significantly enriched hallmark pathways. NES, normalized enrichment score; FDR, false discovery rate. F–J: Heatmaps of gene set variation analysis scores for biological pathways significantly associated with the expression of each

Mφ-DEG. Columns and rows represent individual samples and gene sets, respectively. The high- and low-expression groups are divided by the median value of each Mφ-DEG.

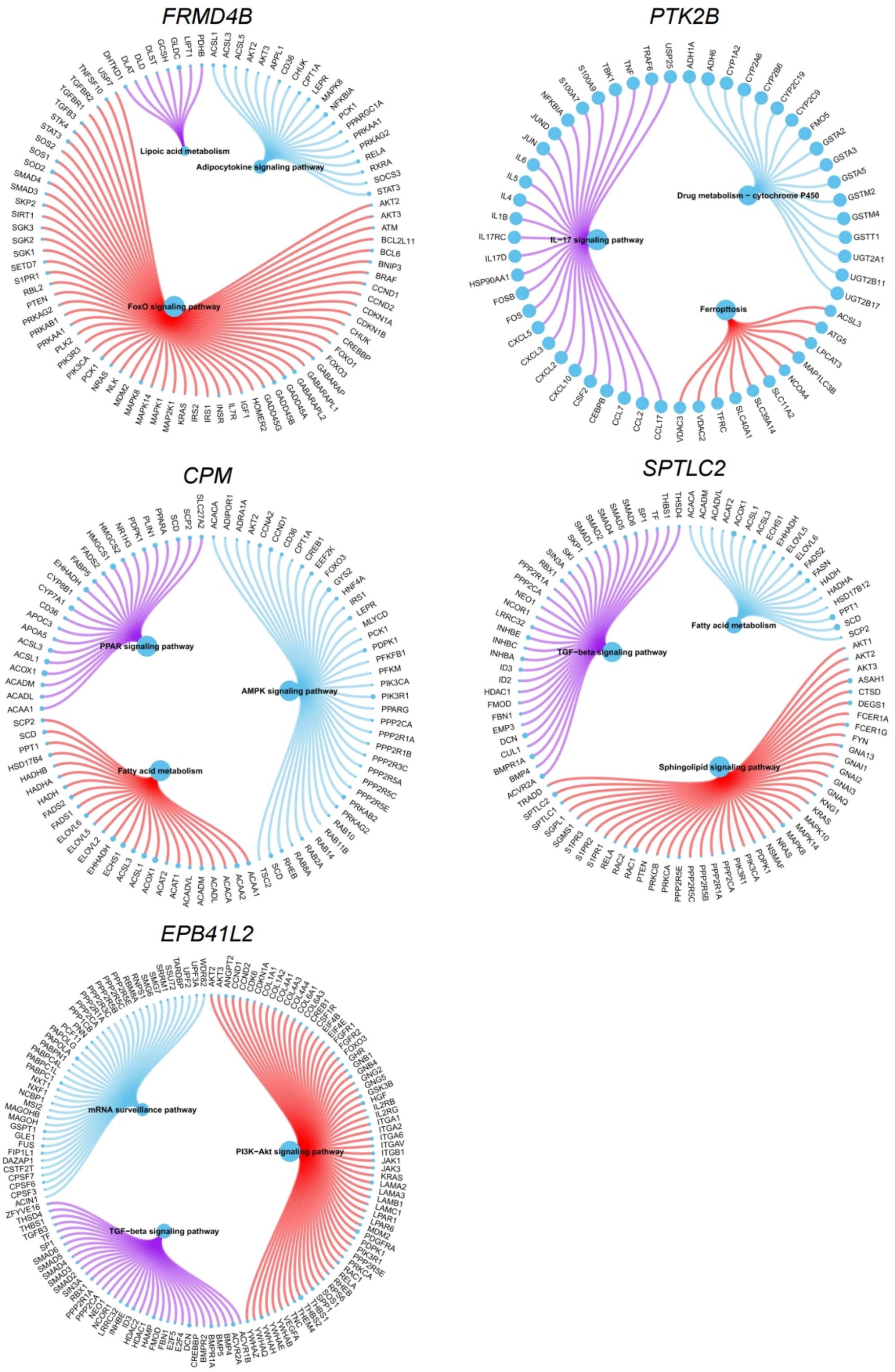


**Fig. S5. Representative gene set enrichment analysis (GSEA) plots for the five key signaling pathways most significantly associated with the five macrophage-related differentially expressed genes (Mφ-DEGs) in metabolic dysfunction-associated steatohepatitis (MASH).**

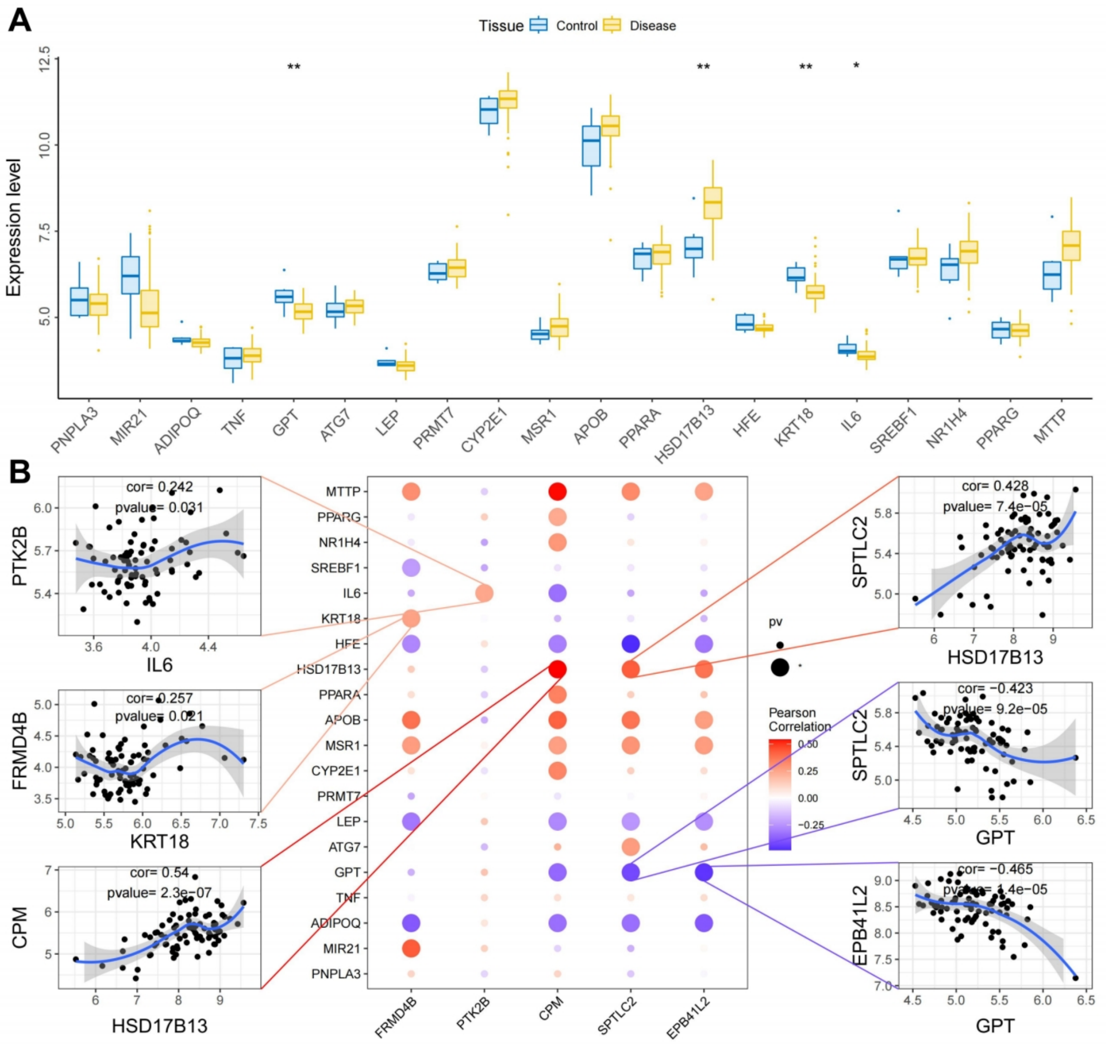


**Fig. S6. Association of macrophage-related differentially expressed genes (Mφ-DEGs) with known metabolic dysfunction-associated steatohepatitis (MASH)-pathogenic genes.** A: Box plots showing the expression of the top 20 MASH-related pathogenic genes (prioritized by GeneCards relevance score) in control (blue) versus disease (yellow) groups from the GSE164760 dataset. B: Correlation heatmap between the expression of the five Mφ-DEGs and the differentially expressed MASH-pathogenic genes. Significant Pearson correlation coefficients (r) are indicated by asterisks and color intensity. $^*P < 0.05$, $^{**}P < 0.01$.

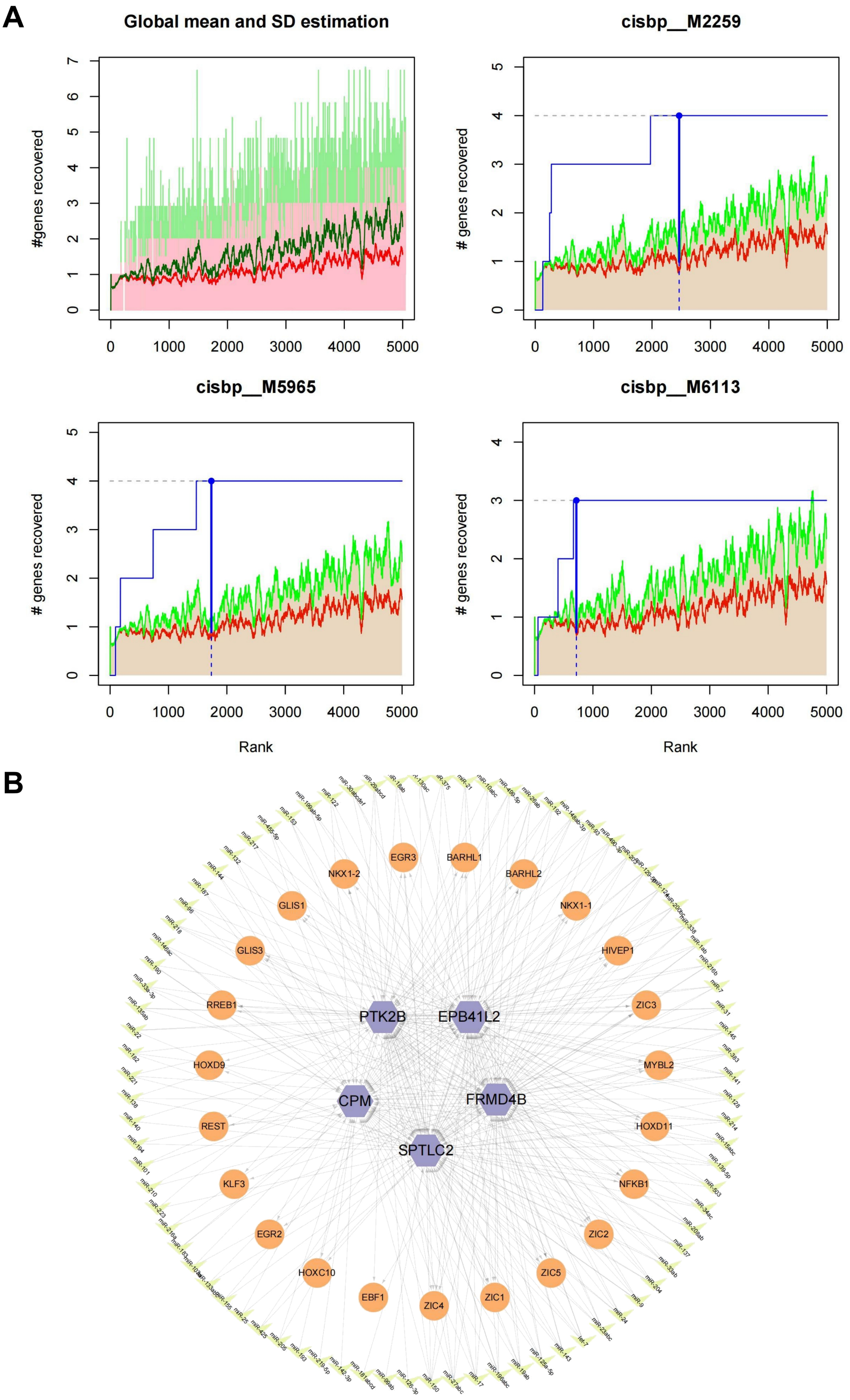


**Fig. S7. Transcriptional regulatory network analysis of macrophage-related differentially expressed genes (Mφ-DEGs). A.** Cumulative recovery curves from transcription factor (TF) enrichment analysis (RcisTarget). The top enriched motif, cisbp__M2259, is highlighted. NES, normalized enrichment score. ***B.*** A comprehensive TF–miRNA–mRNA regulatory network

constructed using predictions from the Cistrome DB and miRcode databases. Triangles represent TFs, circles represent miRNAs, and diamonds represent the five Mφ-DEGs.

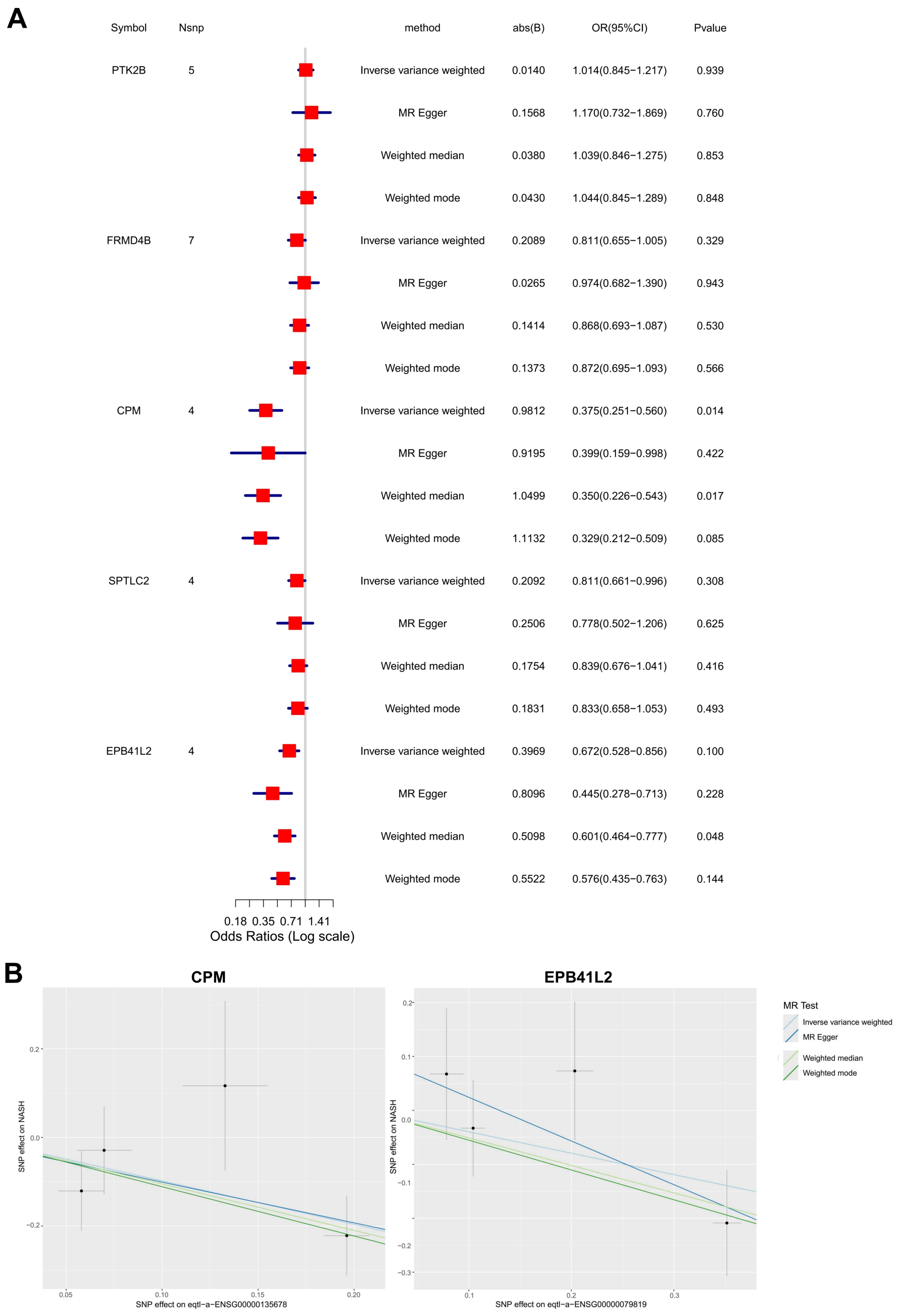


**Fig. S8. Mendelian randomization (MR) analysis of the causal effects of macrophage-related differentially expressed genes (Mφ-DEGs) on metabolic dysfunction-associated steatohepatitis (MASH) Risk.** ***A.*** Forest plots for Mφ-DEGs display the causal estimate (odds ratio, OR) and 95%

confidence interval for each individual single-nucleotide polymorphism (SNP) used as an instrumental variable. ***B.*** Scatter plots for *CPM* (left) and *EPB41L2* (right) illustrate the association between each SNP's effect on gene expression (x-axis) and its effect on MASH risk (y-axis). The slope of the fitted line represents the MR causal estimate from different methods.
