# Supplementary material for: Unveiling novel macrophage-specific biomarkers in MASH through single-cell sequencing for diagnostic modeling
Source: J Lipid Res. 2026 Apr 28;67(6):101048. doi: 10.1016/j.jlr.2026.101048 (PMC13226250; doi:10.1016/j.jlr.2026.101048)
Supplement: Table S1 [file mmc2.docx]

**Supplementary Table 1. Sequences for real-time polymerase chain reaction.**

| Gene | 5'-Sense-3' | 5'-antisense-3' |
| --- | --- | --- |
| *mβ-actin* | TTGTCATCAACCAACTCAGGCTCAG | AGCACTTGGAGGTCAGCATTCTTC |
| *mFRMD4B* | CAGTTCATGGACACCAGGCATTC | TGCTGTAGGCATTCCGAGTCAG |
| *mPTK2B* | CTGGAGAGCATCAACTGTGTGC | GATGGGTAGACGTGTCACAGAG |
| *mCPM* | ATGGAGGCGTTCCTAAAGAGCG | CTCTGTGTTCCTTTGGAGTCTGC |
| *mSPTLC2* | CCAGACTGTCAGGAGCAACCAT | CTTCTTGTCCGAGGCTGACCAT |
| *mEPB41L2* | GGAGGAAAAGGTGTCAGAACTGC | CTTGGCATGGTGCAGGTCAACA |
| *hβ-actin* | GAAGATCAAGATCATTGCTCCTC | ATCCACATCTGCTGGAAGG |
| *hFRMD4B* | ACGCCAGTTCTTACCCGAAACG | TATCGCTGTCCATCTCGGAGAG |
| *hPTK2B* | CATCGTGAAGCTGATCGGCATC | TCTTGTTCCGCTCCAGGTAGTG |
| *hCPM* | CCCAGATGGATTTGAAGCCGTC | CCACAGTTTCAGGCTGCCTTGA |
| *hSPTLC2* | CCAGACTGTCAGGAGCAACCAT | TTCGTGTCCGAGGCTGACCATA |
| *hEPB41L2* | GTAAACGGGTCTCCAGGAGTCT | ACCACGGCAATGCTGACAAGTC |
